# The Red River Cart Model: a Métis conceptualization of health and well-being in the context of HIV and other STBBI

**DOI:** 10.17269/s41997-023-00771-8

**Published:** 2023-04-18

**Authors:** Danielle Atkinson, Rachel Landy, Raye St. Denys, Kandace Ogilvie, Carrielynn Lund, Catherine Worthington, the DRUM & SASH team

**Affiliations:** 1 Faculty of Medicine, University of British Columbia, Vancouver, BC Canada; 2 School of Public Health and Social Policy, University of Victoria, PO Box 1700 STN CSC, Victoria, BC V8W 2Y2 Canada; 3 Shining Mountains Living Community Services, Red Deer, AB Canada; 4 Communities, Alliances, and Networks (CAAN), Fort Qu’Appelle, SK Canada

**Keywords:** HIV, Indigenous, Social determinants of health, Sexually transmitted diseases, Community-based participatory research, VIH, autochtones, déterminants sociaux de la santé, maladies sexuellement transmissibles, recherche participative communautaire

## Abstract

**Objectives:**

Métis people experience health inequities and often face discrimination when accessing health services. Métis-specific services are limited, and pan-Indigenous approaches to health services fail to acknowledge heterogenous identities and distinct health needs of the Métis. This study explored a Métis response to HIV and other sexually transmitted and blood borne infections to inform public health services development for Métis people.

**Methods:**

As part of the DRUM & SASH Project, this study used a community-based research approach which privileged Métis knowledges and processes. Three gathering circles were held in Alberta, Canada, with self-identified Métis individuals who had lived experience or intimate knowledge of HIV/hepatitis C or worked in HIV/HCV service provision. The gathering circle process integrated Métis cultural practices in discussions about Métis understandings of health. Gathering circles transcripts were used to inform the description of the model emerging through the dialogue.

**Results:**

Twelve diverse Métis people participated in gathering circles. Participants identified 12 determinants of health and well-being grounded in Métis culture and imagery, including Métis medicine bag, fiddle, cart tarp, flag, Capote coat, sash, York boat, moccasins, grub box, weapons, tools, and stove. The Red River Cart Model, a Métis-specific model of health to guide service planning, was created from these discussions.

**Conclusion:**

The Red River Cart Model provides a holistic view of the determinants of Métis health and has potential as a collaborative client assessment resource for STBBI community health service providers. Additionally, this model may be helpful to other health service providers for developing Métis-specific/informed services and improving cultural safety for the Métis.

## Résumé

**Objectifs:**

Les Métis font face à des iniquités en santé et sont souvent l’objet de discrimination dans leur accès aux services de santé. Les services centrés sur les Métis sont limités, et les approches panautochtones face aux services de santé ne reconnaissent ni l’hétérogénéité des identités, ni les besoins de santé particuliers des Métis. Notre étude a exploré une intervention métisse face au VIH et à d’autres infections transmissibles sexuellement et par le sang (ITSS) pour éclairer l’élaboration de services de santé publique pour les Métis.

**Méthode:**

Dans le cadre du projet DRUM & SASH, notre étude a utilisé une méthode de recherche de proximité qui a privilégié les savoirs et les processus métis. Trois cercles de rassemblement ont eu lieu en Alberta, au Canada, avec des personnes s’identifiant comme étant métisses et ayant un vécu ou une connaissance intime du VIH ou de l’hépatite C ou ayant travaillé à offrir des services liés au VIH ou au VHC. Le processus des cercles de rassemblement a intégré des pratiques culturelles métisses dans les discussions des savoirs métis en matière de santé. Les transcriptions des cercles de rassemblement ont éclairé la description du modèle ayant émergé de ce dialogue.

**Résultats:**

Douze personnes métisses d’horizons divers ont participé aux cercles de rassemblement. Elles ont défini 12 déterminants de la santé et du bien-être ancrés dans la culture et les images métisses : le sac de guérisseur, le violon, la bâche de charrette, le drapeau, le caban, la ceinture fléchée, la barge d’York, les mocassins, le coffre de nourriture, les armes, les outils et la cuisinière. Le modèle Red River Cart (« charrette de la rivière Rouge »), un modèle spécifiquement métis de la santé, a été créé à partir de ces discussions pour guider la planification des services.

**Conclusion:**

Le modèle Red River Cart présente un portrait holistique des déterminants de la santé des Métis et pourrait être une ressource collaborative d’évaluation des patientes et des patients pour les prestataires de services de santé de proximité liés aux ITSS. Ce modèle pourrait aussi être utile à d’autres prestataires de services de santé pour élaborer des services centrés sur les Métis ou éclairés par la culture métisse et pour améliorer la sécurisation culturelle pour les Métis.

## Introduction

The Métis are one of three legally recognized Indigenous Peoples in Canada, accounting for approximately one third of the country’s Indigenous population (Monchalin & Bourassa, 2019). Métis people arose primarily in the prairies from unions between European fur traders and Indigenous women in the eighteenth century, and have their own unique culture and identity which distinguishes them from First Nation and Inuit Peoples (Macdougall, 2017). Métis people are known for their floral beadwork and embroidery which adorn their garments and traditional items, as well as the colorful Métis sash. In Canada, Métis people experience health disparities, which stem from impacts of colonization including the Scrip system,^1^ exclusion from non-Insured health benefits and other federal policies and programs, and inequitable and culturally unsafe health care services (Monchalin et al., 2019). Health disparities experienced by Indigenous Peoples, including the Métis, extend to those related to human immunodeficiency virus (HIV), hepatitis C (HCV), and other sexually transmitted and blood borne infections (STBBI) (Monchalin & Bourassa, 2019).

In 2018, approximately 62,050 individuals were living with HIV in Canada, with 1 in 8 positive individuals undiagnosed (Public Health Agency of Canada, 2020). In 2017, of the reported HIV cases with a known ethnicity (nationally, only 49.3% of reported HIV cases had an attached ethnic identifier), 20.1% were reported as Indigenous (First Nation, 17.4%; Métis, 2.3%; Inuit, 0.2%; and Indigenous unspecified, 0.3%) (Haddad et al., 2018). An estimated one in ten people living with HIV in Canada is Indigenous, although the lack of historic and/or current ethnic identifiers and differences in provincial reporting practices presents a challenge to both disaggregating the data according to Indigenous identity and estimating the incidence or prevalence of HIV among Métis accurately (Haddad et al., 2018).

Indigenous Peoples in Canada are also overrepresented in rates of other STBBI, although the lack of ethnic identifiers and reporting differences mean that data specific to the Métis are unavailable. In Canada, an estimated 220,697 to 245,987 persons live with hepatitis C (HCV) (approximately 44% undiagnosed) and the rate of HCV within Indigenous populations is estimated to be nearly 5 times higher than in non-Indigenous populations in Canada (Trubnikov et al., 2014). Rates of syphilis have increased significantly since 2010 across Canada, prompting some provinces, such as Alberta, to declare outbreaks (Government of Alberta, 2019).

To reduce rates of HIV infection and other STBBI, effective public health strategies and services are needed, including for testing, education, and treatment. However, for many Indigenous Peoples, including the Métis, research has highlighted limited access to culturally safe health services, i.e., those services that set culturally competent practitioner interactions within a setting that is sensitive to power imbalances, institutional discrimination, and the impact of colonization (Baba, 2013; NAHO, 2008; Monchalin et al., 2022). Existing mainstream approaches to services in the health system focus on individual-level interventions based upon the biomedical model, which are inadequate in addressing the health needs of the Métis (Auger, 2019). Métis-specific research has emphasized the importance of providing culturally safe care specific to Métis peoples in order to improve access to health care (Landy et al., 2022; Monchalin et al., 2019, 2020, 2022). Similar to other Indigenous groups, the Métis conceptualize health in a holistic and multidimensional way which extends beyond the well-being of the individual. Importantly, the Métis share culture, values, and practices that are distinct from other Indigenous groups (Dyck, 2009). The unique culture and identity of the Métis has been identified as a core component of Métis health (Monchalin et al., 2020). Several models of holistic wellness grounded in First Nations cultures have been described in the literature; however, little literature has discussed Métis conceptualizations of health and well-being, which is a knowledge gap acknowledged by Métis and public health researchers (Kumar et al., 2012). While existing research has focused on topics of culturally safe care or defining Métis health and well-being from a physical or mental perspective, there is a lack of research addressing Métis culturally safe care within the realm of HIV/STBBI care, or Métis conceptualizations of health and well-being for those living with or affected by HIV/STBBI (Landy et al., 2022).

Given the need for further work in Métis conceptualizations of health and well-being, and the overrepresentation of Indigenous Peoples (including the Métis) in rates of HIV/STBBI in Canada, this study sought to explore and describe a culturally grounded Métis response to HIV and other STBBI.

## Methods

This study arose from a collaboration between DRUM & SASH (a Canadian Institutes of Health Research (CIHR)–funded team grant that aims to support the development and implementation of community and culturally led interventions to address HIV, HCV, and other STBBI and related mental health issues) and Shining Mountains Living Community Services (Shining Mountains), at the request of the Métis Nation of Alberta (MNA). Shining Mountains is an Indigenous-run agency located in Central Alberta offering a wide variety of health and social services to Indigenous Peoples across the province (Shining Mountains Living Community Services, n.d.). To our knowledge, Shining Mountains is the only community-based agency offering Métis-specific services in Alberta. Ethics approval for this study was provided by University of Victoria’s Human Research Ethics Board (# 18-1179).

### Research approach

Using a community-based research (CBR) approach that privileged Métis knowledge and ways of conducting research, this study aimed to equitably involve Métis community members in the entire research process in order to enhance understandings of the health issues, and improve integration of research findings into the community (Israel et al., 2010). CBR is an approach to research that values collaboration and co-learning among research partners, challenges power imbalances within research, and aims to produce action-oriented research products that benefit those involved (Israel et al., 2010; Minkler, 2005; Wallerstein & Duran, 2008). Through its collaborative approach, CBR can help to decolonize the research process by fully engaging research partners and by privileging Indigenous Knowledges and values within the research context. Partners on this study included Métis and non-Indigenous researchers and Métis community members involved in community development. Specifically, the four study facilitation team members (DA, RSD, KO, & CL) guiding the development process for the conceptual framework are Métis women: DA has mixed Scottish and Cree-Métis ancestry from Duck Lake, Saskatchewan, and the historic Red River Settlement, and resides in Victoria, BC, where she is a second-year medical student. RSD & KO are members of Métis Nation of Alberta and community partners on this work. CL is a member of Gift Lake Métis Settlement located in northern Alberta and currently lives, works, and plays on Treaty 6 territory, the traditional homeland of the Métis and home to many Inuit.

RL and CW have worked in allyship with Métis and First Nation community partners on several studies, including this study and the DRUM & SASH team grant within which it is situated: RL is 2nd-generation Canadian of European descent and CW is of mixed European descent.

The research question and methods were co-developed with academic and community research partners. The development of trust through building relationships was integral to this work and is a core component of Métis approaches to research (LaVallee et al., 2016). This project partnership and approach has been further described in other literature (Landy et al., 2022).

In light of the lack of Métis-specific research regarding culturally safe HIV/STBBI care, and in recognition of the intrinsic link between Métis culture and health, this study sought to explore the question, “What does a Métis-specific cultural response to HIV/STBBI look like?” This question guided data generation about determinants of health specific to Métis who live with HIV/STBBI.

### Sampling and recruitment

A purposive sampling method was used by community-based research partners to identify potential participants. Participants were identified and invited to participate by email, telephone, and in person by staff at Shining Mountains based on their Métis identity and their connections to the Métis community and/or connections to the work of Shining Mountains, and at least one of the following three characteristics: (a) lived experience with HIV/STBBI, (b) experience as an HIV/STBBI service provider, and/or (c) cultural knowledge relevant to Métis health and well-being.

Eight people were recruited to participate with the 4 members of the study facilitation team (the team members were included as both participants and researchers). In total, 12 self-identifying Métis people participated as research participants. They ranged in age from mid 30s to late 70s. They included 8 women, 1 man, and 3 two-spirit-identifying people. Two participants were identified by the community as Elders; 3 were Cree-Michif language consultants. Some participants could only attend a single gathering circle, while others attended all three.

### Data generation

In keeping with the project’s CBR approach, Métis project stakeholders determined that gathering circles (similar to focus groups but with incorporated Métis practices) were an appropriate method to explore the guiding question (Landy et al., 2022). Métis co-authors DA and CL guided the gathering circles, and RSD and KO were present and observed and participated in the discussions. Local protocols and practices were followed throughout the process, including inviting a Métis Elder to be present/participate and gifting of tobacco, to ensure this work was done in a culturally respectful way (Flicker et al., 2015; LaVallee et al., 2016).

The invited Elder began and closed each gathering circle following their local community protocols (e.g., prayer, sharing a teaching). Participants provided verbal informed consent prior to engaging in each gathering circle. Participants were provided with food and refreshments, and an honorarium according to Shining Mountains’ policy. Reciprocity is an important aspect of Métis ways of conducting research; thus, gifts and sharing of food and drink throughout the day were important to building relationships and establishing a supportive environment conducive to this work (LaVallee et al., 2016).

The gathering circles took place over 3 days, each lasting approximately 4 to 7 h. The gathering circles were informally structured around the guiding question “What does a Métis-specific cultural response to HIV/STBBI look like?”, and progressed from identifying Métis determinants of health and well-being to creating a conceptual model of Métis health and well-being grounded in Métis cultural imagery. Once participants decided that a useful outcome for their work would be a model, they discussed what imagery would best illustrate these concepts. Participants identified the Red River Cart as the most appropriate symbol of Métis culture and identity for symbolizing a conceptual model of Métis well-being in the context of HIV/STBBI. Along with the Red River Cart imagery, participants described the items traditionally placed in a Red River Cart as having symbolic meanings related to determinants of well-being needed for a metaphorical journey to well-being. A rough draft of the Red River Cart Model including the items traditionally packed within along with their symbolic relationships related to well-being was developed through discussion during the first gathering circle and further refined in the second gathering circle. A flexible and iterative process was used, allowing participants to flow through topics holistically. Although the model development took place in English, during the final (third) gathering circle, participants determined that it was important to include Cree-Michif nomenclature and further revised the model. Cree-Michif is a traditional Métis language.

### Data analysis

Gathering circles were audio recorded and transcribed by the first author. As the process unfolded, the goal of data analysis became to document the decision-making process of participants, to elaborate upon the rationale for each model component, and to link the data to Métis conceptualizations of health and well-being. Data were managed in NVivo 12 software to assist with coding (QSR International Pty Ltd, 2018). A holistic coding approach was first implemented, which is described by Saldaña (2013) as a process of applying “a single code to each large unit of data in the corpus to capture a sense of the overall contents and the possible categories that may develop” (p. 118). This technique was primarily used because it allowed for the categorizing of entire phenomena which best reflected components of the model and would allow for future, more minute analysis if necessary. Further, it could allow the data from participants to speak for themselves with as little manipulation as possible. Thematic analysis was conducted, which involved reviewing the transcripts and coding the transcripts based on the model components to provide a detailed description of the model and its components, and rationale for component selection and corresponding imagery (Padgett, 2012; Saldaña, 2013; Sundler et al., 2019). Findings were member checked by study team members who were participants at the workshop out of respect for and awareness of research fatigue within the community and were reported back to the community in the form of a report.

## Results

**Fig. 1:**
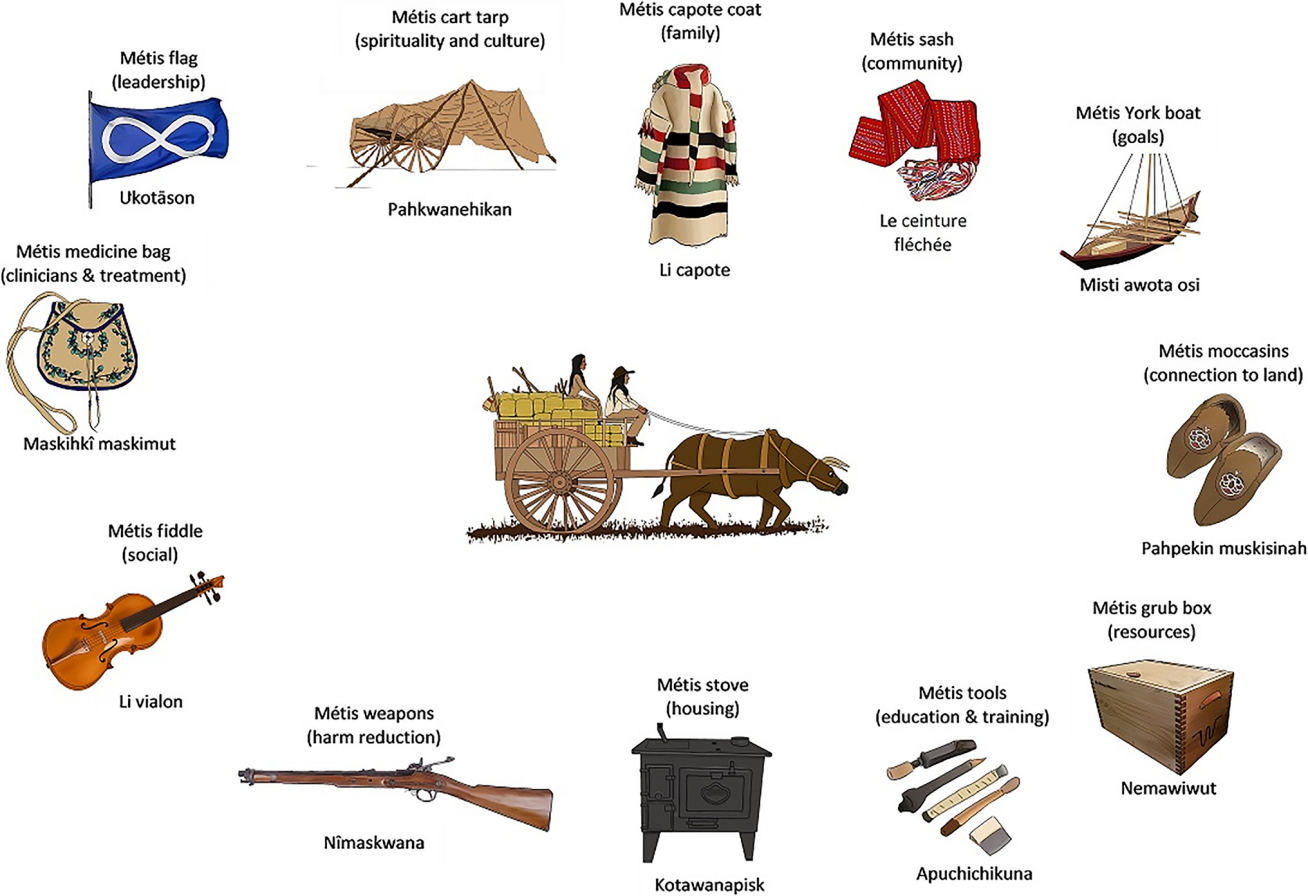
“The Red River Cart Model of Métis Health and Well-being” © Shining Mountains Living Community Services. Adapted with permission. This figure was developed through the research process and copyright is held for a subsequent version of the model by the research partners at Shining Mountains Living Community Services (co-authors RSD and KO) who have given permission for this material to be used in this publication

Through the dialogue in the gathering circles, The Red River Cart Model (RRCM) of Métis Health and Well-being emerged (see Fig. 1). While dialogue was initially centred around HIV/STBBI, the Red River Cart Model emerged as a broader model of determinants of health of those living with or at risk for HIV/STBBI. The Red River Cart is a unique symbol of the Métis, and it has a central role in Métis history and culture. Traditionally semi-nomadic, the Métis packed their most important belongings necessary for survival on the Prairies into the cart. Once the Red River Cart was chosen as a central symbol/image for this model of Métis health and well-being, individual determinants of Métis health were identified by participants and expanded upon through discussions around what one would need to pack for a metaphorical journey to well-being. Participants’ discussions connected each determinant of Métis health and well-being to Métis-specific imagery. Identified components are described in the following section. Where appropriate and based on the available data, quotes are used to illuminate how these discussions supported the identification and description of each model component within a Métis understanding of health and well-being, and detail how each component comprises an important aspect of Métis culture and imagery. Gatherings were also used as opportunities for Elders to share teachings, and in some cases, these were incorporated into the model.

### The Red River Cart Model: determinants of Métis health and well-being

#### The Métis sash (Pukwāhtehon / la ceinture fléchée)—community (māmāwi e āyāk – “living together”)

The Métis sash is traditionally finger-woven and had many purposes: they were used as tools, belts, or rope, and the threads could be pulled out to repair clothing. In the Red River Cart Model, the sash represents the Métis community, while recognizing that the term “community” can mean different things to different people. For instance, community may be centred around volunteering, mentoring, work, spirituality, or culture, but fundamentally participants described community as being about mutual support, with one participant likening the close-knit nature of community to the woven nature of the sash:

Participant 1^2^*: Because that’s kind of where we come from, we were close knit, small communities that traveled through Canadian wilderness, and we took care of each other.*

Participant 2: *I’m a rock collector. And I look at people like rocks. That all of us are like little bitty individual pebbles. And any of them can be smushed, you know, with not a lot of effort. Hammers…we can be destroyed. But if we gather together, like family, we’re a bigger rock and it takes more to destroy that. It can still be done. And if we gather our rocks together we’re a community. And we become a bigger rock. But when we take all of those rocks together we’re a nation. And we’re a country. And nothing destroys us. And to me, the sash is the same. Because it’s made of individual little strands that we can pull them out and break them easy enough. But when we start weaving them together in a braid, or bringing them together, in our family, each time we add to that we’re stronger. Until we have our sash and nothing breaks that. It needs to be strong enough to haul massive loads. And save people’s lives and I think it still does that.*

#### Métis York boat (misti awota osi)—goals (kasiwepahk – “seasonal”)

Since their ethnogenesis, the Métis adapted tools from both their First Nations and their European ancestors. One of these tools was the York boat, a large boat which was used to travel down rivers and portage across land. The York boat was seen by participants as essential for someone on a journey to well-being representing the pursuit of one’s goals. These goals may be related to the betterment of one’s self, family, community, or nation. Participants acknowledged that having goals encouraged them to continually move forward, as is illustrated in the following dialogue:

Participant 1: *When I spoke to the Elders about transition [from traditional to contemporary ways of life], what they said to me is what the biggest barrier is, is that people no longer dream. They have no longer a vision of being somewhere other of whatever they’re in. So, I wonder if the [York] boat could be where the dream is. So, do you have a dream, if you could float this [York] boat somewhere other than where you are, where would you go?*

Participant 2: *Right? And for some of them it’s just a house [place to live].*

Participant 1: *Whatever, whatever. But at least it’s out there.*

Participant 2:* And then that goes to it too because that dream is going to take work on both your parts to get to that dream.*

Participant 1: *Well and it’s something that motivates.*

Participant 2: *It’s a goal. Yeah, it’s a goal.*

#### Métis moccasins (pahpekin muskisinah)—connection to land (āniskohtāw askiy)

Moccasins are shoes made from leather hide and often decorated with Métis beadwork. Participants discussed how moccasins represent connection to the land, since they provide a sole that exists between the feet and the Earth. In Métis culture and worldview, the land feeds, cares for, and heals all. Many Métis rely on nature to improve their health, find comfort, feed their family, or reduce their stress:

*Something that I found healing was when the Elders took me out on the land. Just being with Elders on the land is a whole whack of medicine, and even now if I feel really, really, really overwhelmed, I’ll drop into the grass and just feel connected and remind myself that this is much, much bigger than me. So, I guess just being on the land, I guess you would call it. Just the connection.*

#### Métis grub box (nemawiwut)—resources (nemawiwin)

When packing the Red River Cart, the Métis would place food items inside a wooden box known as the “grub box.” On a metaphorical journey to well-being, resources would be placed in the grub box that would assist with the journey. These might include food banks, transportation subsidies, and other community/social programs:

Participant 1: *So, we learned from Elder [name] that the grub box was a wooden box that was put in the cart and it held all the food stuffs, so through our conversation last time that we had, we thought that would be a good way to represent resources.*

Participant 2: *So, if someone was food insecure, if they had issues paying for their food or getting enough to eat, would that go under: resources or somewhere else? Like using a resource like a food bank?*

Participant 3:* Sounds like something that would be put under resources, like if we think about all the resources a person might use.*

Participant 4: *And there would be bus tickets and stuff under there too.*

#### Métis tools (apuchichikuna)—education and training (kiskinamahowsin atoskewin)

The Métis had specific tools that helped them live a semi-nomadic life. Participants identified education and training to be tools individuals can use on their journey to health and well-being. These tools can include education and training opportunities that help the Métis to improve their socio-economic status and find meaningful employment. This may require attending college or university or joining an apprenticeship program.

Participant 1: *What other education training [would be included here]? Dried blood spot training. You’ve got biomedical training and disease specific training.*

Participant 2:* Cultural training.*

Participant 3: *Shining Mountains does education for all sorts of stuff.*

Participant 2:* Culture and HIV 101…*

#### Métis stove (kotawanapisk – “place where you make fire”)—housing (waskahikana)

The Métis stove was described as a heavy cast-iron stove, which was mobile and used both for heating and cooking. This image was used to represent the need for stable, safe, and affordable housing.

Participant 1: *Sometimes they’re [the individual] looking at getting their housing before they’re looking at spirituality, you know they might be getting their immediate needs met sometimes.*

Participant 2: *Yeah, it all depends on their hierarchy of needs… Because if you have someone who has just been diagnosed and they don’t have housing, really, spirituality and working with that is not going to be as important as trying to find somewhere to be safe and live.*

#### Métis weapons (nîmaskwana)—harm reduction (kaya kitimasohk – “don’t harm yourself”)

Weapons were important for self-protection of Métis families, especially against wildlife. The Métis also used traps and snares to get food and fur hides. This image was chosen to describe health protection as it relates to harm reduction. In the context of HIV and STBBI prevention and care, there are many forms of harm reduction, including supervised consumption sites, family planning and STBBI prevention programs, PrEP/PEP (pre-exposure and post-exposure prophylaxis) for HIV prevention, drug testing, and more.

*I was going to mention that one of the things I haven’t heard is weapons and protection, because back in the day they’d be going into the forest, so they’d need protection, so they would have like an arsenal of different types of weapons and traps, and like snares and what not.*

#### Métis fiddle (li vialon)—social (nukiskātowin)

The Métis fiddle represents the social dimension as a component of health and well-being. Music is the spirit of Métis culture, and is considered healing, as it can provide support and strength during challenging times. Music is prominent in Métis culture as it brings people together to share stories, meals, and dances (called jigs). This determinant of health acknowledges the powerful and prominent role of social activities in the health and well-being of the Métis.

*I was always asked to do music [at a social event], because music can be healing and stuff, so I was wondering if we didn’t want to put a fiddle into the picture as part of the thing [model], or at least put it into the cart.*

#### Métis medicine bag (maskihkî maskimut)—clinicians and treatment (nātawihowin – “to feel better”)

Medicine bags carry items or ingredients that have healing power. This could be traditional medicines like sage or sweetgrass, medicinal herbs like mint or ratroot, or items with special meaning or significance like a feather or a stone. Métis worldviews acknowledge the healing power of both Western medicine and traditional medicine in the maintenance of one’s health and well-being, and therefore adopt a holistic definition of treatment, inclusive of allied health care as well. One participant described the contents of medicine bags:

*I have a bag that… it’s been in the family forever. Um, so I guess I have two. One was a smaller pouch that, um, could hold medicines. Small. Like whether it was tobacco or, um, sage or mint or whatever, that got put in there… I have no idea how old they are. The smaller bag is beaded and the other is made with fur and leather.*

*But my understanding was that we had medicines and we had moss that was used for bandages and if we got hurt, threads could be taken out of our sash to stitch people up.*

#### Métis flag (ukotāson – “thing you raise up”)—leadership (nîkaniw)

The Métis Nation has many strong leaders and advocates for the Métis and their rights. The Métis flag represents the support individuals (and service providers) receive from those in leadership positions in the Métis community.

*Now we have the flag which honours the importance of our political leaders and the flag that holds our people together under one Peoples that recognizes us as a nation… the flag that we need our political leaders in the wagon with us to support the work [to address HIV/STBBI in Métis communities] we are doing, to help in that.*

#### Métis cart tarp (pahkwanehikan)—our spirituality and culture (tapokeh tamowin – “the truth” / nistam pimatoowin – “culture/your first life”)

The Métis cart tarp, made of canvas or hide, was placed over the contents of the cart. It protected the contents from the harsh prairie weather. Participants described the role of Métis culture and spirituality as essential to protecting and promoting their health and well-being.

Participant 1: *Does culture belong up in the top with spirituality or does it belong down with the social? Is it more of a social thing or is it a covering that should stay with the tarp? That would be one question.*

Participant 2:* I would say it should stay. Spirituality and culture are so intertwined.*

Participant 3: *Culture is spirituality, and spirituality is culture.*

Participants acknowledged that there are diverse expressions of Métis culture and spirituality. For instance, many Métis people pray for protection and guidance from the Creator or ancestors, while others attend formal churches of various denominations. Additionally, Elders have a large role in Métis spirituality and culture as knowledge-holders and language keepers. Participants also recognized that not everyone is religious or spiritual, and that many Métis individuals are not raised in their culture. However, they also described that many people feel a spiritual connection to something greater than themselves, like nature, or humanity, or may feel drawn to a specific cause that is very important to them. In these ways, this determinant also represents spirituality and culture for those who do not engage in more formal practices.

#### Métis capote coat (li capote / miskotakay)—family (peyakosan / li famille)

The capote was a coat traditionally worn by Métis fur traders. It was made out of iconic wool trading blankets and was tied at the waist with a belt or sash. The capote wraps around an individual to keep them warm and safe, which was described by participants as a metaphor for the essential role of family in health and well-being. The participants adopted a holistic and broad understanding of the term “family”, acknowledging that family can take many forms and is not limited to one’s “family of creation”.

*It [the Métis capote coat] represents a kinship, the non judgemental, the warm, and compassionate services, the care of each other, taking care of each other.*

## Discussion

The participants of this study used the image of the Red River Cart as the central image in this model of Métis health and well-being in the context of HIV and other STBBI. As described above, the Red River Cart holds a place of great cultural significance for the Métis who are a traditionally nomadic People and the Cart is often associated with Métis identity. While the participants of this study conceptualized a model of health that positioned the determinants of health as items of cultural significance that one would pack into the Red River Cart for a journey to health and well-being in this context, others have also used Red River Cart imagery in their conceptualizations of Métis well-being. For instance, Sakitawak Métis Nation, Ile-à-la-Crosse (Askiy Consulting Inc., 2005) used the image of the Red River Cart wheel in their conceptualization of Métis community wellness, and the Métis Nation-Saskatchewan Health Department has adapted this model to present their conceptualization of Métis well-being, showing the interconnectedness of dimensions of health including language, culture, history, and spirituality using the wheel’s hub, spokes, and felloes (LaVallee, 2014).

While the Government of Canada recognizes 12 social determinants of health as affecting all people, the unique experiences of Indigenous Peoples and the health disparities they face call for the need to identify the unique social determinants of Indigenous Peoples’ health (Government of Canada, 2019). Some social determinants of health specific to Indigenous Peoples have been identified, including: experiencing systemic racism; a lack of cultural safety within the healthcare system; community infrastructure, resources and capacity; the health of the environment; cultural continuity; colonialism; and lack of self-determination at the level of individuals, communities, and nations (Loppie Reading & Wien, 2009). The participants of this study identified 12 determinants specific to Métis health and well-being: family, community, goals, connection to land, resources, education and training, housing, harm reduction, social, employment, clinicians and treatment, spirituality and culture, and leadership. These determinants are congruent with those identified by others, such as Dyck and the Métis Centre at the National Aboriginal Health Organization (2009), who indicated that Métis-specific determinants of health are strongly linked to the historical, socio-political and economic contexts of the Métis. Dyck and colleagues (2009) included as determinants of Métis health: marginalization; denial of Métis rights and a lack of recognition of Métis identity; the removal of Métis lands and rights via anti-Métis policies; and the subsequent loss of Métis culture, knowledge, languages, and spirituality. Further, they suggest that one should not fail to acknowledge factors such as resiliency, healing, and resurgence, which have been integral to Métis survival and well-being (Dyck, 2009). In our study, the participants identified determinants of Métis health and well-being focused on promoting health, resiliency, healing, and resurgence, rather than factors associated with ill health.

### Improving health services and culturally safe access to care for the Métis

Across Canada, the Métis face a lack of recognition and services across multiple health and social sectors (Auger, 2019; Dyck, 2009; Evans et al., 2012; Monchalin, 2019; Monchalin & Bourassa, 2019; Monchalin et al., 2022). Exclusion from federally funded, supported, and administered health programs has had long-standing negative impacts on the health and well-being of the Métis and has served to perpetuate existing health inequities (Auger, 2019; Smylie, 2009; Wesche, 2013). The lack of Métis-specific service provision means that most Métis seek care from mainstream services, which can be culturally unsafe (Wesche, 2013). While most Métis communities desire the ability to determine and provide their own health services, communities are often underfunded (Evans et al., 2012), and lack the necessary resources (whether financial, human, knowledge, or governmental support) or Métis-specific educational resources, models, or approaches to care. The Red River Cart Model provides a preliminary framework through which the development of community-grounded Métis-specific health interventions can begin to occur.^3^

Research has identified that service providers often have a limited knowledge or understanding of Métis culture, identity, and issues, which can reduce the comfort of individuals seeking care (Wesche, 2013). An overarching lack of Métis-specific research means that Métis-specific conceptualizations, understandings or models of health and well-being are not widespread within academic literature (Kumar et al., 2012). This lack of Métis-specific conceptualizations or models of health has contributed to inadequate integration of Métis perspectives in health policy and services and an overall lack of understanding of the unique health and social needs of the Métis (Auger, 2019). The Red River Cart Model provides a visual, easy-to-understand, concrete example of some of the ways in which the Métis experience and understand their own health and well-being. Thus, it fills a gap that exists in health policy, service provision, and academic literature.

The Red River Cart Model presents a holistic model of Métis health and well-being for individuals working in Indigenous health or public health who may have limited knowledge or understanding of Métis culture as a determinant of Métis health. This conceptual model has many implications for policy makers and health care providers. While some of the model components overlap with other well-researched determinants of health (i.e., housing), others require more thought in their application within policy (i.e., importance of connection to land in health and well-being) and how Métis community members or governments can be engaged with respect to these determinants. However, the application of this model is not limited to use within policy; potential uses include use as a discussion or planning tool within a care provider/social service provider – client/patient relationship. This model was developed with the intention for it to be built upon as a future service model as well as within policy contexts in the future. This model is shared with the intention that its use as a theoretical framework can improve public health awareness and knowledge of Métis culture and Métis-specific determinants of health, and can inform the development, evaluation, and improvement of policy and services towards the Métis.

## Conclusion

Aiming to increase the knowledge of Métis health and well-being through an empowering process of community consultation and gathering-circle discussion, this study sought to explore Métis conceptualizations of health resulting in the creation of a model of health and well-being developed by Métis, for Métis. Developed specifically for HIV and other STBBI, it can be applied in many contexts. The Red River Cart Model offers public health policy makers with a concrete tool, grounded in Métis culture, which can support the planning and delivery of Métis-specific/informed public policy and health services. This model is offered as a way for practitioners to increase their understanding of the Métis health and well-being needs, as well as an opportunity for Métis communities to work towards developing or advocating for health and well-being services for the Métis according to the needs of their specific communities.

## Contributions to knowledge

What does this study add to existing knowledge?

- This study offers a conceptual model of Métis health and well-being developed in the context of prevention, education, treatment, and care of HIV and other STBBI.
- This study used empowering processes—by Métis, for Métis—to guide the development of Métis-specific resources.

What are the key implications for public health interventions, practice, or policy?

- The Métis have a unique culture and determinants of well-being that are distinct from those of other Indigenous Peoples.
- Public health services and policies addressing Indigenous Peoples must acknowledge the unique determinants of well-being for the Métis, and where/when available, Métis-specific services should be provided.

## Data Availability

N/A.

## Footnotes

1 The Scrip System, implemented by the colonial government in 1870, was used “to divide and administer the land promised to the Métis. Land allotments arranged through Scrip were unsuitable for agriculture, and prevented Métis solidarity and resistance” (Monchalin et al., 2020, p. 328).

2 Where more than one participant is speaking, the voices have been indicated with numbers.

3 This model has already been adapted and implemented as an assessment tool by staff and clients at Shining Mountains.

## Code availability

N/A.
